# The Hospitals Association and Its Seceding Offshoot

**Published:** 1888-03-17

**Authors:** K. W. Peter

**Affiliations:** Lady Superintendent. Jenny Lind Infirmary for Sick Children, 86, Pottergate Street, Norwich


### THE HOSPITALS ASSOCIATION AND ITS SECEDING OFFSHOOT.

As I agree with "H. M." that it is unadvisable to start a number of small or rival associations for nurses, I should be glad of information as to why the British Nurses' Association has been formed. It cannot be intended to clash with the Hospitals Association, as I see several ladies are members of both. Is the British Nurses' Association in connection with the Hospitals Association? or if not, what is the special work which they intend to take up that has not already been taken up by the Hospitals Association?

K. W. PETER, Lady Superintendent.

Jenny Lind Infirmary for Sick Children,
86, Pottergate Street, Norwich.

[The object of the British Nurses' Association is to promote the obtaining of a charter for the registration of nurses. The Hospitals Association is an institution of much wider scope. It has not only a registration scheme on hand, but has founded the National Pension Fund, which is now a *fait accompli*, and is devoted to the advancement of every branch of hospital administration.—ED. *T. H.*]

